# Examining abnormal Silurian trilobites from the Llandovery of Australia

**DOI:** 10.7717/peerj.14308

**Published:** 2022-11-04

**Authors:** Russell D.C. Bicknell, Patrick M. Smith

**Affiliations:** 1 Palaeoscience Research Centre, School of Environmental and Rural Science, University of New England, Armidale, New South Wales, Australia; 2 Department of Biological Sciences, Macquarie University, Sydney, New South Wales, Australia; 3 Palaeontology Department, Australian Museum Research Institute, Sydney, New South Wales, Australia

**Keywords:** Abnormalities, Trilobites, Paleozoic, Teratology, Silurian, *Odontopleura (Sinespinaspis) markhami*

## Abstract

Abnormal trilobites present insight into how arthropods with fully biomineralised exoskeletons recovered from injuries, genetic malfunctions, and pathologies. Records of abnormal Silurian trilobites in particular show an abundance of specimens with teratologies and a limited record of injuries. Here we expand the record of abnormal Silurian trilobites by presenting seven new abnormal specimens of *Odontopleura (Sinespinaspis) markhami* from the early Silurian (Llandovery, Telychian) Cotton Formation, New South Wales. We use these specimens to illustrate novel evidence for asymmetric distribution of pleural thoracic spine bases. These abnormal bases likely reflect genetic complications, resulting in morphologies that would unlikely have aided the fitness of abnormal individuals. In considering records of malformed Silurian trilobites more broadly, we propose that the largest trilobites may have been prey at this time. This indicates a possible change in the trophic position of trilobites when compared to Cambrian and Ordovician palaeoecosystems.

## Introduction

Abnormal extinct organisms allow for predator–prey interactions, genetic malfunctions, and injury recovery to be assessed in fossil groups (Owen, 1985; Babcock, 1993a; Babcock, 2003; Babcock, 2007; Kelley, Kowalewski & Hansen, 2003; Huntley, 2007; Klompmaker & Boxshall, 2015; Leung, 2017). Due to the palaeobiological importance of these specimens, abnormalities have been documented in many fossil groups (Klompmaker et al., 2019). Euarthropods, in particular, have been documented showing injuries (Owen, 1985; Bicknell & Paterson, 2018), pathologies (Lochman, 1941; Šnajdr, 1978b), and teratologies (Pocock, 1974; Lee, Choi & Pratt, 2001; Bicknell & Smith, 2021). While abnormalities are known from arachnids (Mitov, Dunlop & Bartel, 2021), crustaceans (Bishop, 1972; Klompmaker et al., 2013; Klompmaker et al., 2014), and horseshoe crabs (Bicknell, Pates & Botton, 2018), the most well documented abnormal euarthropods are trilobites (Šnajdr, 1978a; Owen, 1983; Owen, 1985; Babcock, 1993a; Babcock, 2003; Fatka, Budil & Grigar, 2015; Fatka, Budil & Zicha, 2021; Bicknell, Paterson & Hopkins, 2019; Bicknell & Holland, 2020; Zong, 2021). The detailed record of trilobite abnormalities is due to the biomineralised dorsal exoskeleton exhibited by the group, a structure that increases the preservational potential of specimens and readily permits the record of abnormal structures. Trilobites are, therefore, an ideal group for understanding how a wholly extinct clade of euarthropods experienced and recovered from abnormalities.

**Figure 1:**
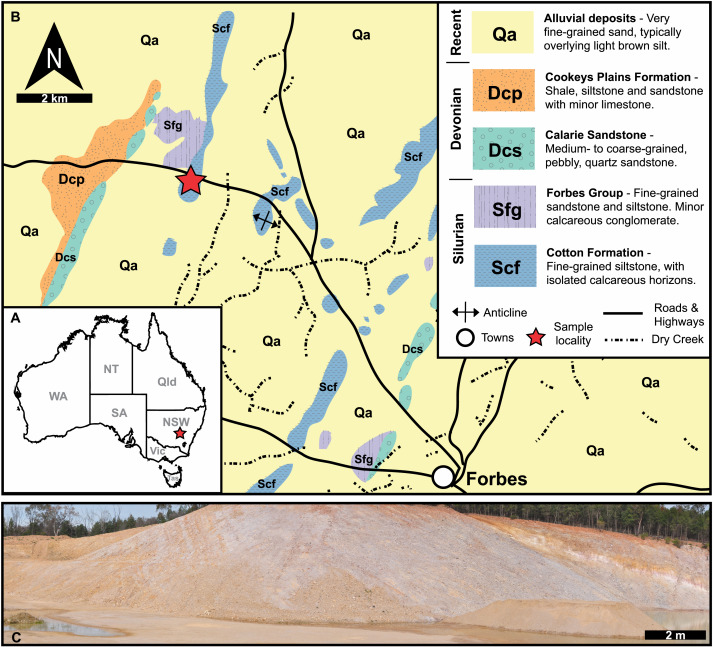
Geological, stratigraphic, and geographical information for specimen locations and the Cotton Hill Formation. (A) Map of Australia showing specimen location (red star) in New South Wales. (B) Geological map showing rocks proximal to Forbes. Red stars indicate specimen location. (C) Panoramic view of located where specimens were collected–Cotton Hill Quarry.

**Figure 2:**
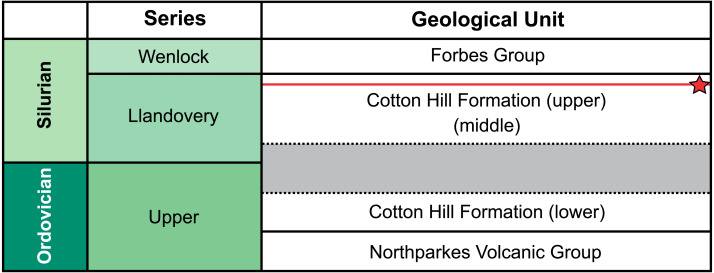
Correlation of selected Late Ordovician and Silurian rock units surrounding the Cotton Formation within the Forbes area. Approximate position of sampled trilobite horizon indicated by red line and star symbol. Grey section indicates time break between the lower and two upper members (Percival & Glen, 2007).

A large number of documented abnormal trilobite specimens are from Cambrian-aged deposits (*e.g.*, Owen, 1985; Babcock, 1993a; Babcock, 2003; Pates et al., 2017; Pates & Bicknell, 2019; Bicknell & Pates, 2020; Zong, 2021). These specimens commonly record failed predation (Rudkin, 1979; Babcock, 1993a; Bicknell & Paterson, 2018), and show limited evidence for genetic or teratological complications (see Bergström & Levi-Setti, 1978; Bicknell et al., 2022a). By contrast, the record of abnormal post-Cambrian trilobites shows developmental malformations, teratologies, and pathologies, with fewer injuries derived from predation (*e.g.*, Owen, 1985; Rudkin, 1985; Zong, 2021; Bicknell et al., 2022c). Silurian-aged deposits in particular preserve a diverse array of abnormal taxa across at least ten families (Table 1). These abnormalities primarily reflect developmental malfunctions (Šnajdr, 1981a; Bicknell & Smith, 2021), injuries and abnormal recovery from moulting (Šnajdr, 1981a), with rarer evidence for failed attacks (Chinnici & Smith, 2015; Bicknell, Paterson & Hopkins, 2019) and accidental trauma (Rudkin, 1985). These specimens also present insight into how the occasionally ornate, often iso- to macropygous, Silurian taxa recovered from moulting and developmental complications. Historically, most abnormal Silurian trilobites are reported from deposits in the Czech Republic (*e.g.*, Přibyl & Vaněk, 1962; Přibyl & Vaněk, 1986; Šnajdr, 1976; Šnajdr, 1978a; Šnajdr, 1978b; Šnajdr, 1979; Šnajdr, 1980; Šnajdr, 1981a; Šnajdr, 1981b), Sweden (*e.g.*, Ramsköld, 1983; Ramsköld, 1984; Owen, 1985; Ramsköld et al., 1994), and the USA (*e.g.*, Campbell, 1967; Whittington & Campbell, 1967; Holloway, 1980; Rudkin, 1985; Whiteley, Kloc & Brett, 2002; Chinnici & Smith, 2015; Bicknell, Paterson & Hopkins, 2019). However, more recent records of abnormal Silurian trilobites from Australia (Bicknell & Smith, 2021) and China (Zong et al., 2017; Zong, 2021) suggest a more Gondwanan presence of these abnormal specimens. This indicates that abnormal trilobites from middle Paleozoic may have a much more global record than previously thought. To expand this line of enquiry, here we considered the trilobite-rich Cotton Formation, central New South Wales (NSW) and illustrate new examples of abnormal odontopleurids (Edgecombe & Sherwin, 2001; Rickards, Wright & Thomas, 2009; Figs. 1 and 2).

**Table 1:** Record of abnormal Silurian trilobites. Ordered by stage and then genus.

Taxon	Family	Series	Stage	Formation, country	Abnormality location	Abnormality description	Side	Citation and figure
*Acernaspis elliptifrons* (Esmark, 1833)	Lichidae	Llandovery	Aeronian	Solvik Formation, Sweden	Pygidium	Asymmetrically developed furrows	Both	Owen (1985, fig. 5t)
*Encrinurus squarrosus* Howells, 1982	Encrinuridae	Llandovery	Aeronian	Newlands Formation, Scotland	Pygidium	Damaged rib	Right	Howells (1982, pl. 8, fig. 12)
*Encrinurus squarrosus*	Encrinuridae	Llandovery	Aeronian	Newlands Formation, Scotland	Pygidium	Bifurcating rib	Right	Howells (1982, pl. 8, fig. 13)
*Coronocephalus* sp.	Encrinuridae	Llandovery	Telychian	Fentou Formation, China	Pygidium	Deformed, fused pygidial ribs	Right	Zong (2021, fig. 4D, E)
*Coronocephalus* sp.	Encrinuridae	Llandovery	Telychian	Fentou Formation, China	Pygidium	Truncated pygidial ribs	Right	Zong et al. (2017, fig. 3q); Zong 2021, fig. 4F, G)
*Coronocephalus* sp.	Encrinuridae	Llandovery	Telychian	Fentou Formation, China	Pygidium	Additional pygidial rib	Right	Zong (2021, fig. 4H, I)
*Kailia intersulcata* (Chang, 1974)	Encrinuridae	Llandovery	Telychian	Fentou Formation, China	Thorax	Thoracic spines 2–5 truncated, U-shaped indentation	Right	Zong (2021, fig. 4A–C)
*Odontopleura* (*Sinespinaspis*) *markhami*	Odontopleuridae	Llandovery	Telychian	Cotton Formation, NSW, Australia	Thorax	Additional thoracic spine base	Right	This article, Figs. 3A and 3B
*Odontopleura* (*Sinespinaspis*) *markhami*	Odontopleuridae	Llandovery	Telychian	Cotton Formation, NSW, Australia	Thorax	Additional spine base and offset spine base	Right	This article, Figs. 3C and 3D
*Odontopleura* (*Sinespinaspis*) *markhami*	Odontopleuridae	Llandovery	Telychian	Cotton Formation, NSW, Australia	Thorax	Additional posterior pleural band spine bases	Right	This article, Figs. 4A and 4B
*Odontopleura* (*Sinespinaspis*) *markhami*	Odontopleuridae	Llandovery	Telychian	Cotton Formation, NSW, Australia	Thorax	Additional thoracic spine base	Right	This article Figs. 4C and 4D
*Odontopleura* (*Sinespinaspis*) *markhami*	Odontopleuridae	Llandovery	Telychian	Cotton Formation, NSW, Australia	Thorax	Additional thoracic spine base	Right	This article, Figs. 4E and 4F
*Odontopleura* (*Sinespinaspis*) *markhami*	Odontopleuridae	Llandovery	Telychian	Cotton Formation, NSW, Australia	Thorax	Additional thoracic spine base	Right	This article, Figs. 5A and 5B
*Odontopleura* (*Sinespinaspis) markhami*	Odontopleuridae	Llandovery	Telychian	Cotton Formation, NSW, Australia	Thorax	Additional posterior pleural band spine bases	Left	This article, Figs. 5C and 5D
*Decoroproetus corycoeus* (Conrad, 1842)	Proetidae	Wenlock	Sheinwoodian- Homerian	St. Clair Formation, Arkansas, USA	Thorax, pygidium	Thoracic segment 11? fused to pygidium	Right	Holloway (1980, pl. 3, fig. 4)
*Calymene frontosa* Lindström, 1885	Calymenidae	Wenlock	?Sheinwoodian	Visby Beds, Sweden	Cephalon	Abnormal development of suture	Left	Owen (1985, fig. 5c)
*Arctinurus boltoni* (Bigsby, 1825)	Lichidae	Wenlock	Sheinwoodian	Rochester Formation, New York, USA	Pygidium	Truncated posteriormost pygidial spine, ‘W’-shaped injury	Right	Rudkin (1985, fig. 1A, B)
*Arctinurus boltoni*	Lichidae	Wenlock	Sheinwoodian	Rochester Formation, New York, USA	Thorax, pygidium	Large ‘U’-shaped indentation, posterior thorax, extending onto pygidium	Right	Babcock (1993b, p. 36, no figure number)
*Arctinurus boltoni*	Lichidae	Wenlock	Sheinwoodian	Rochester Formation, New York, USA	Cephalon, thorax, pygidium	‘U’-shaped indentation, cephalon; ‘V’-shaped indentation thoracic segments 3–4; ‘W’-shaped indentation thoracic segments 8–10 ‘U’-shaped indentation pygidium	Left (cephlaon, thorax) Right (pygidium)	Whiteley, Kloc & Brett (2002 fig. 2.9B); Chinnici & Smith (2015, fig. 434)
*Arctinurus boltoni*	Lichidae	Wenlock	Sheinwoodian	Rochester Formation, New York, USA	Thorax, pygidium	Thoracic spines 1–4 truncated, ‘U’-shaped indentation, truncated pygidial spines	Right (thorax) Left (pygidium)	Chinnici & Smith (2015, fig. 432)
*Arctinurus boltoni*	Lichidae	Wenlock	Sheinwoodian	Rochester Formation, New York, USA	Cephalon, thorax	‘U’-shaped indentation, posterior cephalon, single segment injury, 4th thoracic segment	Right	Chinnici & Smith (2015, fig. 433)
*Arctinurus boltoni*	Lichidae	Wenlock	Sheinwoodian	Rochester Formation, New York, USA	Pygidium	Abnormal pygidial spine	Left	Bicknell, Paterson & Hopkins (2019, fig. 3A, B)
*Arctinurus boltoni*	Lichidae	Wenlock	Sheinwoodian	Rochester Formation, New York, USA	Pygidium	Reduced pygidial spine	Right	Bicknell, Paterson & Hopkins (2019, fig. 3C, D)
*Arctinurus boltoni*	Lichidae	Wenlock	Sheinwoodian	Rochester Formation, New York, USA	Pygidium	‘U’-shaped indentation	Right	Bicknell, Paterson & Hopkins (2019, fig. 3E, F)
*Arctinurus boltoni*	Lichidae	Wenlock	Sheinwoodian	Rochester Formation, New York, USA	Pygidium	Rounded pygidial spine	Right	Bicknell, Paterson & Hopkins (2019, fig. 4A, B)
*Arctinurus boltoni*	Lichidae	Wenlock	Sheinwoodian	Rochester Formation, New York, USA	Pygidium	‘W’-shaped indentation	Right	Bicknell, Paterson & Hopkins (2019, fig. 4C, D)
*Arctinurus boltoni*	Lichidae	Wenlock	Sheinwoodian	Rochester Formation, New York, USA	Pygidium	‘W’-shaped indentation	Right	Bicknell, Paterson & Hopkins (2019, fig. 4E, F)
*Arctinurus boltoni*	Lichidae	Wenlock	Sheinwoodian	Rochester Formation, New York, USA	Thorax	Single segment injury, thoracic segment 2	Right	Bicknell, Paterson & Hopkins (2019, fig. 5A, B)
*Arctinurus boltoni*	Lichidae	Wenlock	Sheinwoodian	Rochester Formation, New York, USA	Thorax and pygidium	Two ‘V’-shaped indentations (thoracic segments 1–2; thoracic segments 7–8); pygidium slightly truncated	Right	Bicknell, Paterson & Hopkins (2019, fig. 6A, B)
*Calymene niagarensis* (Hall, 1843)	Calymenidae	Wenlock	Sheinwoodian	Rochester Formation, New York, USA	Thorax	‘L’-shaped indentation, thoracic segments 1–4	Right	Chinnici & Smith (2015, fig. 432)
*Calymene* sp.	Calymenidae	Wenlock	Sheinwoodian	Rochester Formation, New York, USA	Cephalon	Borings on genal spine	Left	Whiteley, Kloc & Brett (2002, fig. 2.15D–F)
*Coronocephalus urbis* Strusz, 1980	Encrinuridae	Wenlock	Sheinwoodian	Walker Volcanics, Australian Central Territory, Australia	Pygidium	Bifurcated rib	Right	Strusz (1980, pl. 1, fig. 17)
*Dalmanites limulurus* (Green, 1832)	Dalmanitidae	Wenlock	Sheinwoodian	Rochester Formation, New York, USA	Thorax	‘U’-shaped indentation, thoracic segments 2–5	Right	Chinnici & Smith (2015, fig. 437)
*Dalmanites limulurus*	Dalmanitidae	Wenlock	Sheinwoodian	Rochester Formation, New York, USA	Thorax	U’-shaped indentation, thoracic segments 1–3	Right	Chinnici & Smith (2015, fig. 438)
*Dalmanites limulurus*	Dalmanitidae	Wenlock	Sheinwoodian	Rochester Formation, New York, USA	Thorax	‘U’-shaped indentations, thoracic segments 2–4 and 8–1	Left	Chinnici & Smith (2015, fig. 439); Whiteley, Kloc & Brett (2002, fig. 2.15A)
*Dalmanites limulurus*	Dalmanitidae	Wenlock	Sheinwoodian	Rochester Formation, New York, USA	Thorax, pygidium	U’-shaped indentation, thoracic segments 10–11 extending into pygidium	Left	Chinnici & Smith (2015, fig. 440)
*Dalmanites limulurus*	Dalmanitidae	Wenlock	Sheinwoodian	Rochester Formation, New York, USA	Thorax	U’-shaped indentation, thoracic segments 5–11	Left	Chinnici & Smith (2015, fig. 441)
*Dalmanites limulurus*	Dalmanitidae	Wenlock	Sheinwoodian	Rochester Formation, New York, USA	Pygidium	Terminal, medial spine missing	Midline	Whiteley, Kloc & Brett (2002, fig. 2.15C)
*Japonoscutellum* sp.	Encrinuridae	Wenlock	Sheinwoodian	Yarralumla Formation, New South Wales, Australia	Pygidium	Bifurcating axial rib	Right	Bicknell & Smith (2021, fig. 3b, c)
*Exallaspis bufo* (Ramsköld, 1984)	Odontopleuridae	Wenlock	Homerian	Mulde Beds, Sweden	Cranidium	Asymmetrical crandium	Left	Ramskold (1984, pl. 31, fig. 1)
*Exallaspis bufo*	Odontopleuridae	Wenlock	Homerian	Mulde Beds, Sweden	Pygidium	Additional terminal spine	Midline	Ramskold (1984, pl. 31, fig. 5)
*Interproetus truncus* Šnajdr, 1980	Proetidae	Wenlock	Homerian	Liten Formation, Czech Republic	Thorax	Reduced and fused pleurae	Right	Šnajdr (1980, pl. XLVIII, figs 1, 2)
*Ktenoura retrospinosa* Lane, 1971	Cheiruridae	Wenlock	Homerian	Much Wenlock Limestone Formation, England	Pygidium	Reduced spine	Right	Lane (1971, pl. 6, fig. 9a, b)
*Odontopleura ovata* Emmrich, 1839	Odontopleuridae	Wenlock	Homerian	Liten Formation, Czech Republic	Thorax	‘U’-shaped indentation, thoracic segments 4–8	Right	Šnajdr (1979, pl. 1)
*Exallaspis mutica* (Emmrich, 1844)	Odontopleuridae	Wenlock–Ludlow	—	Grünlich-Graues Graptolithengestein, Germany	Pygidium	Single spine injury	Left	Šnajdr (1969, pl. IV, fig. 7)
*Odontopleura ovata*	Odontopleuridae	Wenlock–Ludlow	—	Grünlich-Graues Graptolithengestein, Germany	Pygidium	Asymmetric medial lobe	Left	Schrank (1969, pl II, fig. 4)
*Alcymene lindstroemi* Ramsköld et al., 1994	Calymenidae	Ludlow	Gorstian	Hemse Marl, Sweden	Cephalon	Overdeveloped glabellar region	Midline	Ramskold (1994, fig. 5, 9)
*Bohemoharpes ungula viator* Přibyl & Vaněk, 1986	Harpetidae	Ludlow	Gorstian	Kopanina Formation, Czech Republic	Cephalon	Asymmetrical cranidial region	Right larger than left	Přibyl & Vaněk (1986, pl. 2, fig.1)
*Bohemoharpes ungula*	Harpetidae	Ludlow	Gorstian	Kopanina Formation, Czech Republic	Cephalon	Multiple neoplasms	Left	Šnajdr (1978a, pl. I, figs. 1–5)
*Bohemoharpes ungula*	Harpetidae	Ludlow	Gorstian	Kopanina Formation, Czech Republic	Cephalon	Neoplasms on genal spine	Left	Šnajdr (1978a, pl. I, figs. 6, 7); Šnajdr (1990, p. 63)
*Prionopeltis archiaci* (Barrande, 1846)	Proetidae	Ludlow	Gorstian	Kopanina Formation, Czech Republic	Pygidium	Single spine injury	Right	Šnajdr (1981a, pl. I, fig. 1)
*Prionopeltis archiaci*	Proetidae	Ludlow	Gorstian	Kopanina Formation, Czech Republic	Pygidium	‘U’-shaped indentation	Right	Šnajdr (1981a, pl. II, fig. 2)
*Prionopeltis archiaci*	Proetidae	Ludlow	Gorstian	Kopanina Formation, Czech Republic	Pygidium	Fused pygidial ribs, ‘W’-shaped indentation	Right	Šnajdr (1981a, pl V, fig. 4)
*Prionopeltis archiaci*	Proetidae	Ludlow	Gorstian	Kopanina Formation, Czech Republic	Pygidium	Pinched pygidial ribs	Left	Šnajdr (1981a, pl V, fig. 5; pl VIII, fig. 3)
*Prionopeltis archiaci*	Proetidae	Ludlow	Gorstian	Kopanina Formation, Czech Republic	Pygidium	Additional terminal spine	Midline	Šnajdr (1981a, pl VII, fig. 6)
*Prionopeltis archiaci*	Proetidae	Ludlow	Gorstian	Kopanina Formation, Czech Republic	Pygidium	Thin terminal spines	Midline	Šnajdr (1981a, pl VIII, fig. 4)
*Prionopeltis archiaci*	Proetidae	Ludlow	Gorstian	Kopanina Formation, Czech Republic	Pygidium	Ribs poorly developed	Right	Šnajdr (1981a, pl VIII, fig. 5)
*Prionopeltis archiaci*	Proetidae	Ludlow	Gorstian	Kopanina Formation, Czech Republic	Pygidium	Additional spine	midline	Šnajdr (1981a, pl VIII, fig. 6)
*Prionopeltis archiaci*	Proetidae	Ludlow	Gorstian	Kopanina Formation, Czech Republic	Pygidium	Additional spine	Left	Šnajdr (1981a, pl VIII, fig. 7)
*Prionopeltis archiaci*	Proetidae	Ludlow	Gorstian	Kopanina Formation, Czech Republic	Pygidium	Additional spine	Midline	Šnajdr (1981a, pl VIII, fig. 8)
*Prionopeltis dracula* Šnajdr, 1980	Proetidae	Ludlow	Gorstian	Kopanina Formation, Czech Republic	Pygidium	Additional spines	Both	Šnajdr (1980, not figured)
*Scharyia micropyga* (Hawle & Corda, 1847)	Aulacopleuridae	Ludlow	Gorstian	Kopanina Formation, Czech Republic	Pygidium	‘U’-shaped indentation, spine abnormally developed	Right	Šnajdr (1981a, pl IV, fig. 2)
*Scharyia micropyga*	Aulacopleuridae	Ludlow	Gorstian	Kopanina Formation, Czech Republic	Pygidium	Additional ribs	Midline	Šnajdr (1981b, pl. XI, fig. 1)
*Scharyia micropyga*	Aulacopleuridae	Ludlow	Gorstian	Kopanina Formation, Czech Republic	Pygidium	Abnormally developed interring furrows	Midline	Šnajdr (1981b, pl. XI, fig. 2)
*Scharyia micropyga*	Aulacopleuridae	Ludlow	Gorstian	Kopanina Formation, Czech Republic	Pygidium	Abnormally developed interring furrows	Midline	Šnajdr (1981b, pl. XI, fig. 3)
*Scharyia micropyga*	Aulacopleuridae	Ludlow	Gorstian	Kopanina Formation, Czech Republic	Pygidium	Abnormal axial ring	Midline	Šnajdr (1981b, pl. XI, fig. 4)
*Scharyia micropyga*	Aulacopleuridae	Ludlow	Gorstian	Kopanina Formation, Czech Republic	Pygidium	Abnormal axial ring	Midline	Šnajdr (1981b, pl. XI, fig. 7)
*Scharyia micropyga*	Aulacopleuridae	Ludlow	Gorstian	Kopanina Formation, Czech Republic	Pygidium	Poorly developed axial rings	Midline	Šnajdr (1981b, pl. XI, fig. 8)
*Sphaerexochus latifrons* Angelin, 1854	Cheiruridae	Ludlow	Gorstian	Hemse Marl, Sweden	Cephalon	Pathological development on free cheek	Right	Ramsköld (1983, pl. 19, fig. 6)
*Kosovopeltis nebula* Campbell, 1967	Scutelluidae	Ludlow	Gorstian–early Ludfordian	Henryhouse Formation, Oklahoma, USA	Thorax	Overdeveloped pleurae	Right	Campbell (1967, pl. 2 figs 5, 6)
*Batocara robustus* (Mitchell, 1924)	Encrinuridae	Ludlow	Ludfordian	Black Bog Shale, New SouthWales	Thorax	Bifurcated pleural rib	Right	Strusz (1980, pl. 3, fig. 7)
*Batocara robustus*	Encrinuridae	Ludlow	Ludfordian	Black Bog Shale, New South Wales, Australia	Pygidium	Offset axial nodes	Midline	Bicknell & Smith (2021, fig. 2a, b)
*Batocara robustus*	Encrinuridae	Ludlow	Ludfordian	Black Bog Shale, New South Wales, Australia	Pygidium	Bifurcating axial rib	Left	Bicknell & Smith (2021, fig. 2c, f
*Batocara robustus*	Encrinuridae	Ludlow	Ludfordian	Black Bog Shale, New South Wales, Australia	Pygidium	Additional axial node	Midline	Bicknell & Smith (2021, fig. 2d, e)
*Didrepanon squarrosum*	Cheiruridae	Ludlow	Ludfordian	Kopanina Formation, Czech Republic	Crandium	Asymmetric glabellar furrows	Left	Přibyl & Vaněk (1973, pl. I, fig. 1)
*Leonaspis rattei* (Etheridge & Mitchell, 1869)	Odontopleuridae	Ludlow	Ludfordian	Black Bog Shale, New South Wales, Australia	Thorax	Asymmetrical thoracic pleural spine base	Both	Bicknell & Smith (2021, fig. 3a)
*Harpidella* (*Rhinotarion*)*setosum*Whittington & Campbell, 1967	Aulacopleuridae	Ludlow	?Ludfordian	Hardwood Mountain Formation, Maine, USA	Cephalon	Asymmetrical cranidium	Left larger than right	Whittington & Campbell (1967, pl. 5, fig. 5, 6)
*Prionopeltis striata* Barrande, 1846	Proetidae	Pridoli	—	Přídolí Formation, Czech Republic	Pygidium	Single spine injury	Left	Šnajdr (1981a, pl. I, fig. 2)
*Prionopeltis striata*	Proetidae	Pridoli	—	Přídolí Formation, Czech Republic	Pygidium	‘W’-shaped indentation	Left	Šnajdr Šnajdr (1981a), pl. I, fig. 3)
*Prionopeltis striata*	Proetidae	Pridoli	—	Přídolí Formation, Czech Republic	Pygidium	Spines removed	Left	Šnajdr (1981a, pl. II, fig. 3)
*Prionopeltis striata*	Proetidae	Pridoli	—	Přídolí Formation, Czech Republic	Pygidium	‘V’-shaped indentation	Right	Šnajdr (1981a, pl. II, fig. 5)
*Prionopeltis striata*	Proetidae	Pridoli	—	Přídolí Formation, Czech Republic	Pygidium	Fused, deformed ribs	Left	Šnajdr (1981a, pl. III, fig. 1)
*Prionopeltis striata*	Proetidae	Pridoli	—	Přídolí Formation, Czech Republic	Pygidium	‘V’-shaped indentation	Left	Šnajdr (1981a, pl. III, fig. 8)
*Prionopeltis striata*	Proetidae	Pridoli	—	Přídolí Formation, Czech Republic	Cephalon	Shallow ‘U’-shaped indentation in free cheek	Right	Šnajdr (1981a, pl. IV, fig. 5)
*Prionopeltis striata*	Proetidae	Pridoli	—	Přídolí Formation, Czech Republic	Pygidium	Pathological growth	Midline	Šnajdr (1981a, pl. IV, fig. 6); De Baets et al. (2021, fig. 6.2f)
*Prionopeltis striata*	Proetidae	Pridoli	—	Přídolí Formation, Czech Republic	Pygidium	Additional spine, posteriormost section	Midline	Šnajdr (1981a, pl. VII, fig. 2)
*Prionopeltis striata*	Proetidae	Pridoli	—	Přídolí Formation, Czech Republic	Pygidium	‘U’-shaped indentation	Midline	Šnajdr (1981a, pl. VII, fig. 4)
*Prionopeltis striata*	Proetidae	Pridoli	—	Přídolí Formation, Czech Republic	Pygidium	‘U’-shaped indentation	Midline	Šnajdr (1981a, pl. VII, fig. 5)
*Prionopeltis striata*	Proetidae	Pridoli	—	Přídolí Formation, Czech Republic	Pygidium	‘U’-shaped indentation	Midline	Šnajdr (1981a, pl. VIII, fig. 1)
*Prionopeltis striata*	Proetidae	Pridoli	—	Přídolí Formation, Czech Republic	Pygidium	‘W’-shaped indentation	Left	Šnajdr (1981a, pl. VIII, fig. 2)
*Scharyia nympha* Chlupáč, 1971	Aulacopleuridae	Pridoli	—	Přídolí Formation, Czech Republic	Pygidium	Additional ribs, asymmetrically developed	Midline	Šnajdr (1981b, pl. XII, fig. 7)
*Tetinia minuta* (Přibyl & Vaněk, 1962)	Proetidae	Pridoli	—	Přídolí Formation, Czech Republic	Pygidium	Reduced ribs	Right	Šnajdr (1981a, pl. II, fig. 7)
*Tetinia minuta*	Proetidae	Pridoli	—	Přídolí Formation, Czech Republic	Pygidium	‘U’-shaped indentation, pinched ribs	Right	Šnajdr (1981a, pl. II, fig. 8)
*Tetinia minuta*	Proetidae	Pridoli	—	Přídolí Formation, Czech Republic	Pygidium	U’-shaped indentation, abnormal ribs	Left	Šnajdr (1981a, pl. III, fig. 4)
*Tetinia minuta*	Proetidae	Pridoli	—	Přídolí Formation, Czech Republic	Pygidium	Asymmetrical pygidium, abnormal ribs	Left	Šnajdr (1981a, pl. III, fig. 5)
*Tetinia minuta*	Proetidae	Pridoli	—	Přídolí Formation, Czech Republic	Pygidium	Asymmetrical medial lobe, abnormal ribs	Left	Šnajdr (1981a, pl. III, fig. 6)

## Methods

Trilobite specimens from the Cotton Formation housed within the Australian Museum (AM F), Sydney, NSW, Australia were examined under a microscope. Seven abnormal *Odontopleura* (*Sinespinaspis*) *markhami* (Edgecombe & Sherwin, 2001) specimens were identified. These specimens were dyed black with ink, coated in magnesium oxide, and photographed under low angle LED light with a Canon EOS 5DS. An additional 39 standard specimens were also photographed using this equipment. However, as they are not figured, they were not dyed or coated. Images were stacked using Helicon Focus 7 (Helicon Soft Limited) stacking software.

**Figure 3:**
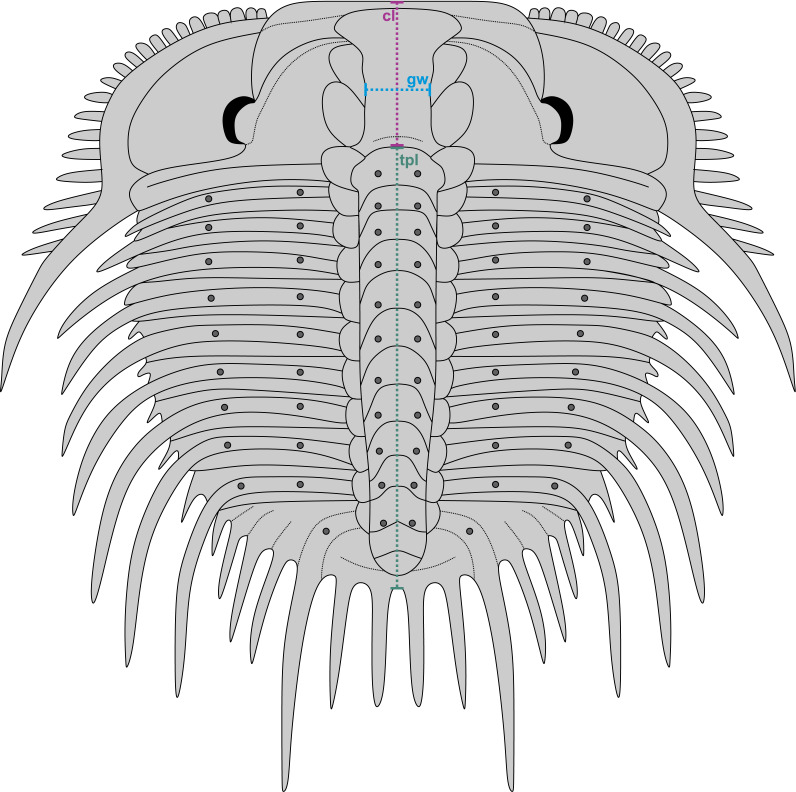
Reconstruction of *Odontopleura* (*Sinespinaspis*) *markhami* showing measurements taken for analysed dataset. Abbreviations: cl, cranidial length; gw, glabellar width; tpl, combined thorax and pygidium length.

A dataset of linear measurements was collated to determine where abnormal *Odontopleura* (*Sinespinaspis*) *markhami* specimens are located relative to standard individuals in bivariate space. Measurements of the cranidial length, glabellar width, and combined thorax and pygidium length were taken from 46 specimens (*n* = 39 normal, *n* = 7 abnormal) in the AM F collection (Fig. 3). The dataset was collated from specimen photographs using ImageJ (Schneider, Rasband & Eliceiri, 2012) (Data S1). Measurements were natural-log normalised and plotted, points were colour coded for presence or absence of abnormalities.

### Geological context

The material reported herein comes from “Cotton Hill Quarry”, at approximately 33°18′44.0″S 147°56′00.9″E, on the western limb of the Forbes Anticline within the Cotton Formation (Fig. 1). The geological context of this site was discussed in detail by Edgecombe & Sherwin (2001, p 87–90). Hence, only a summary is provided here. Generally, the formation outcrops poorly, appearing only as low rubbly hills in the Forbes region. Occasionally it is exposed in road and rail cuttings, as well as locally in gravel quarries. The Cotton Formation at “Cotton Hill Quarry” consists of well-bedded, thinly to moderately laminated siltstone which readily splits along the bedding plane (Fig. 1C). The outcrop varies considerably in colour, mostly being an off-white to light brownish yellow. However, in limited patches, it is deep orange to purple, often associated with large Liesegang rings. The floor of the quarry reveals that the original, unweathered rock is a darker grey colour and contains interbeds of whiter tuff that show signs of small-scale slumping. The quarry walls indicate a dip at 65° to the west and a minimum thickness of 105 m in its upper member. Previous reports suggest the entire Cotton Formation could be up to 1,500 m in total thickness on the eastern limb of the Forbes Anticline (Sherwin, 1973), assuming a consistent dip and no cover.

Traditionally, the entire Cotton Formation was thought to range across the Ordovician—Silurian boundary (Sherwin, 1970; Sherwin, 1973; Fig. 2). However, to date, only three horizons are known to contain age diagnostic graptolite faunas. The oldest of these—the “lower member”—has been assigned a possible Katian (late Ordovician) age. The “middle” and “upper members” contain fauna indicative of early and late Llandovery (early Silurian) age respectively (Sherwin, 1974; Rickards, Wright & Thomas, 2009). So far, there is no conclusive evidence of Hirnantian or earliest Llandovery graptolites, suggesting a significant time break between the “lower member” and the remaining two members in the formation (Percival & Glen, 2007). The material from “Cotton Hill Quarry” is derived from singular horizons within the upper-most 50 m of the formation, typically the “upper member”. Here the trilobites co-occur with a distinct *Spirograptus turriculatus* Zone graptolite fauna. Sherwin (1973, fig. 4) also noted a similar trilobite fauna ∼20 m from the quarry, occurring one meter above beds with the eponym of the graptolite zone. Sherwin also noted the trilobites occurred 100 m stratigraphically above a horizon with *Monograptus* cf. *sedgwicki*. This strongly supports a late Llandovery age for the “Cotton Hill Quarry” material (Edgecombe & Sherwin, 2001).

Variability in lithology of the members has resulted in a variety of depositional environments suggested for the Cotton Formation (*e.g.*, Krynen, Sherwin & Clarke, 1990). The “upper member” exposed at “Cotton Hill Quarry” likely formed in a calm outer-shelf environment, below storm wave base, as evidenced by the well-laminated siltstone and the lack of disarticulated trilobites and echinoderms. The abundant planktonic graptolites and common small-eyed (or blind) trilobite taxa suggest that the environment was relatively deep, limiting light penetration. However, the benthic faunas (*e.g.*, rare dendroidal graptolites, strophomenid brachiopods, platyceratid gastropods, and echinoderms) suggests that the bottom waters were still well-oxygenated.

## Results

Abnormalities on *Odontopleura* (*Sinespinaspis*) *markhami* are minute (sub-millimetre scale) and primarily record the asymmetry of thoracic posterior pleural band spine bases.

**Figure 4:**
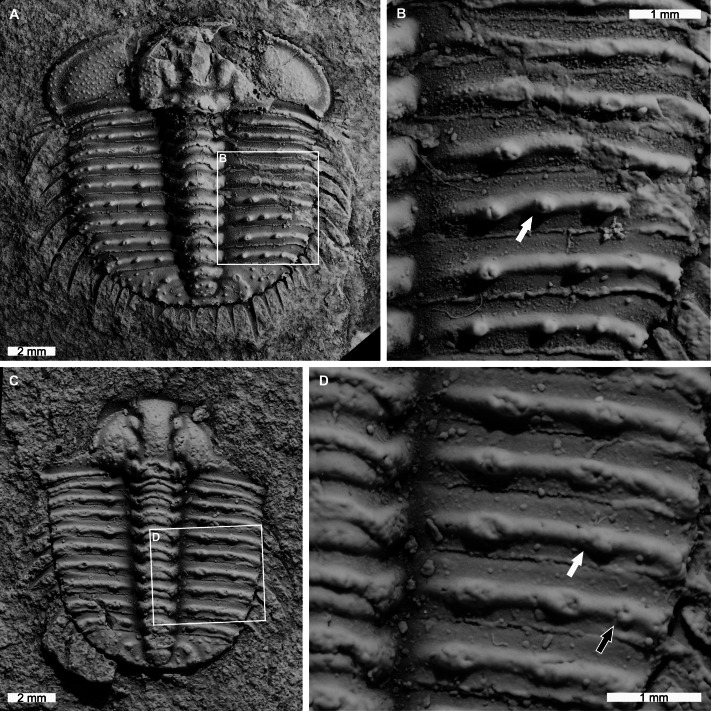
*Odontopleura* (*Sinespinaspis*) *markhami* with additional and abnormal spine bases on the right thoracic lobe. (A, B) AM F126904. (A) Complete specimen. (B) Close up of box in (A) showing additional spine base on the seventh thoracic segment (white arrow). (C, D) AM F118762. (C) Complete specimen. (D) Close up of box in (C) showing offset spine base (white arrow) and additional spine base (black arrow).

AM F126904 is a near complete specimen, 13.3 mm long, 10.3 mm wide (excluding genal and pleural spines) with an asymmetric distribution of thoracic posterior pleural band spine bases (Figs. 4A, 4B). The seventh thoracic segment on the right pleural lobe has an additional spine base when compared to the left side.

AM F118762 is a moult, lacks free cheeks, is 12.2 mm long, 10.2 mm wide (excluding pleural spines) with one offset spine base and one additional spine base on the right pleural lobe (Figs. 4C, 4D). The sixth thoracic segment has an offset spine base and the seventh segment has an additional base.

**Figure 5:**
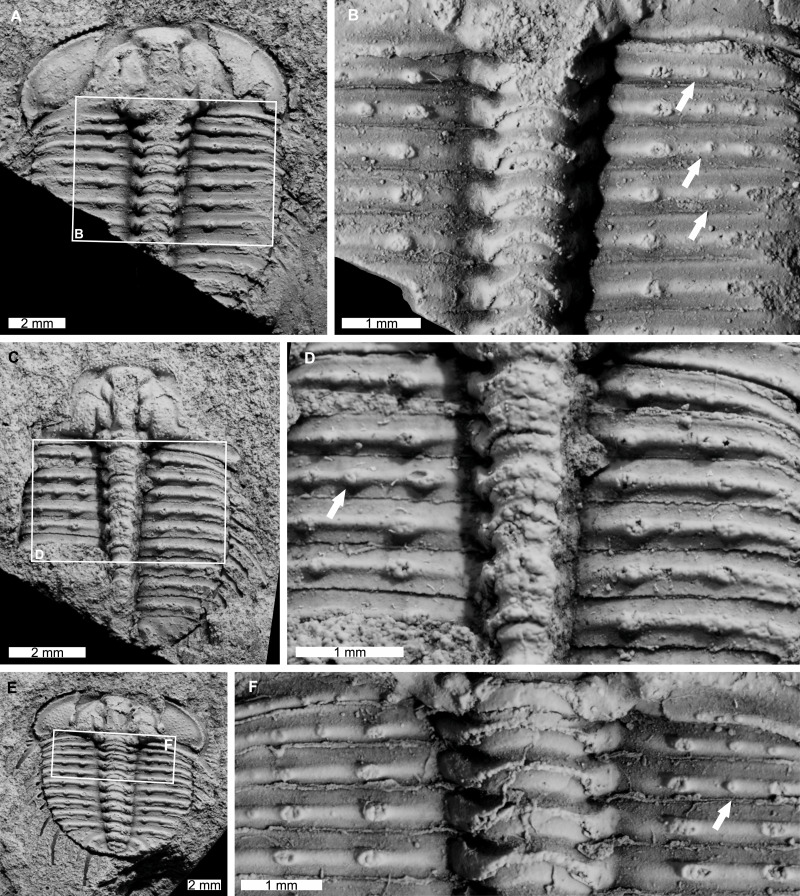
*Odontopleura* (*Sinespinaspis*) *markhami* showing additional spine bases. (A) Complete specimen. (B) Close up of box in (A) showing additional spine bases on first, third, and fourth thoracic segments on the right pleural lobe (white arrows). (C, D) AM F115081. (C) Complete specimen. (D) Close up of box in (C) showing additional spine base on the third thoracic segment of the left pleural lobe (white arrow). (E, F) AM F145135. (E) Complete specimen. (F) Close up of box in (E) showing additional spine bases on second thoracic segment of the right pleural lobe (white arrow).

AM F115089 is a partial specimen, lacks a posterior section, is 13.3 mm long, 12.0 mm wide (excluding pleural and genal spines) with an asymmetrical distribution of thoracic posterior pleural band spine bases (Figs. 5A, 5B). The first, third, and fourth thoracic segments on the right pleural lobe have an additional spine base not observed on the left lobe.

AM F115081 is a partial specimen, lacking the posterior portion of the exoskeleton, likely a moult, is 10.8 mm long, 7.0 mm wide (excluding pleural spines). The specimen has an additional thoracic spine base on the left pleural lobe (Figs. 5C, 5D). The third thoracic segment has an additional base not observed on the right lobe.

AM F145135 is 11.7 mm long, 12.4 mm wide (excluding pleural and genal spines) with an additional thoracic spine base on the right pleural lobe (Figs. 5E, 5F). The second thoracic segment has an additional base not observed on the left lobe.

**Figure 6:**
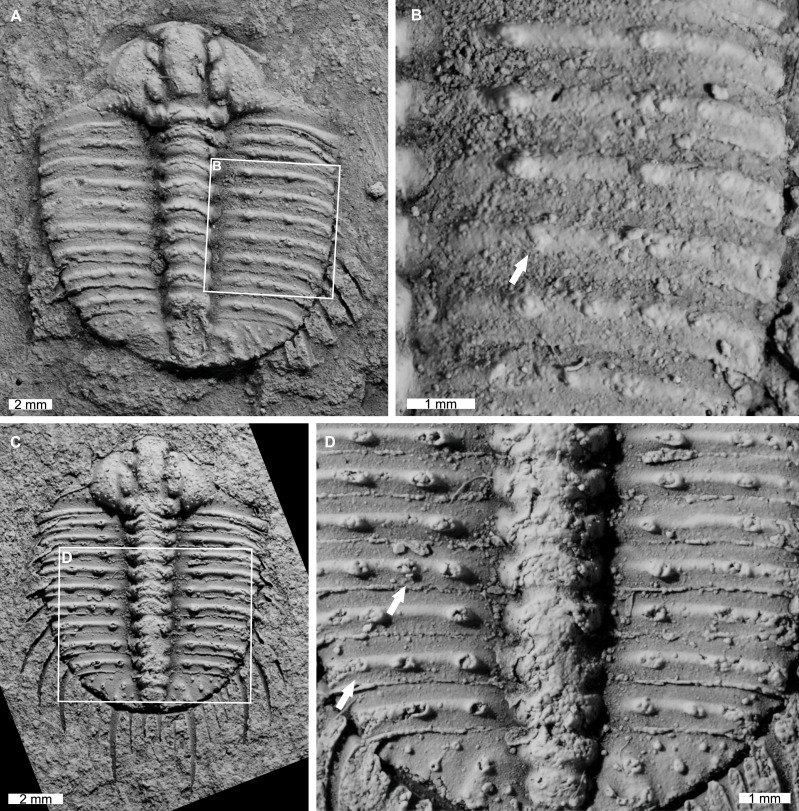
*Odontopleura* (*Sinespinaspis*) *markhami* with additional and offset spine bases. (A, B) AM F118772. (A) Complete specimen. (B) Close up of box in (A) showing offset spine on the sixth thoracic segment of the right pleural lobe (white arrow). (C, D) AM F133034. (C) Complete specimen. (D) Close up of box in (C) showing additional spine bases on the sixth and eighth thoracic segments of the left pleural lobe (white arrows).

AM F118772 is likely a moult, lacks free cheeks, is 14.7 mm long, 12.9 mm wide (excluding pleural spines). The specimen has an abnormal spine base on the right pleural lobe (Figs. 6A, 6B). The sixth thoracic segment has a thoracic spine base unaligned with the immediately anterior and posterior spine bases.

AM F133034 is likely a moult, lacks free cheeks, is 10.7 mm long, 9.1 mm wide (excluding pleural spines). The specimen has an asymmetrical distribution of thoracic pleural spine bases (Figs. 6C, 6D). The sixth and eighth thoracic segments on the left pleural lobe have an additional spine bases not observed on the right lobe.

**Figure 7:**
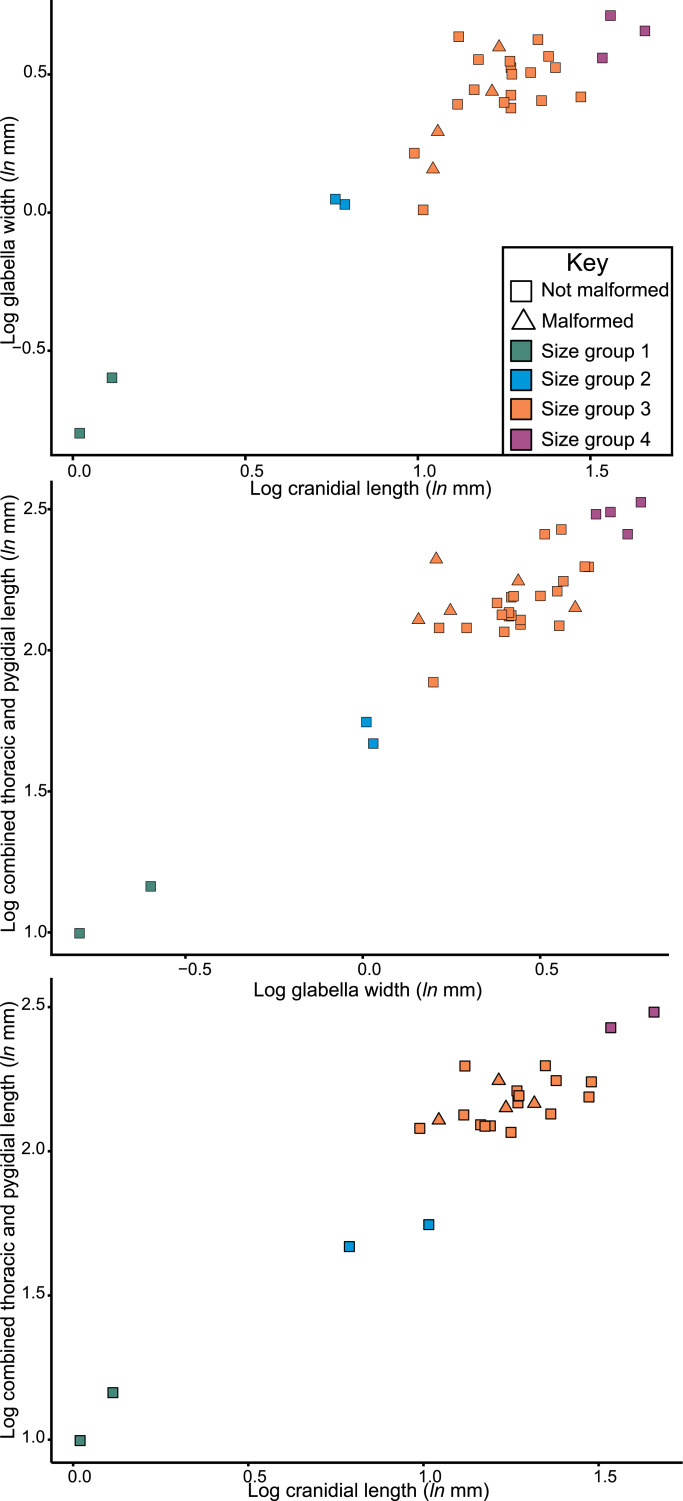
Natural log normalised bivariate plots of *Odontopleura* (*Sinespinaspis*)* markhami* of abnormal and standard specimens. Abnormal specimens are located in size group 3.

Considering the size distribution of *Odontopleura* (*Sinespinaspis*) *markhami* in bivariate space, four distinct clusters are noted (Fig. 7). We propose that four holaspid size groups are documented. The abnormal specimens are located within the second largest observed size grouping.

## Discussion

*Odontopleura* (*Sinespinaspis*) *markhami* abnormalities represent additional thoracic spine base developments or offset of spine bases. Despite the presence of these abnormal structures, there is no evidence for exoskeletal removal, or any other damage to specimens. Therefore, abnormal spine base development does not reflect abnormal recovery from an injury induced during moulting or from a failed attack. These abnormalities must have arisen through another process. In life, odontopleurid trilobites had large spines that preserve as spine bases on internal moulds (Bruton, 1966). Additional spine bases therefore record development of spines that arose outside the primary spine sequences. Such additional spines may have resulted in more effective defence against possible predators. However, the Cotton Formation biota show few predators (Edgecombe & Sherwin, 2001). Furthermore, the spines would not have resulted in an increased reproductive fitness as thoracic spinosity is unlikely to be a sexually selected morphology, unlike cephalic spines (Knell & Fortey, 2005; Knell et al., 2013). Given these conditions, it seems that the additional bases record teratological developments through genetic malfunctions.

Similar additional spine bases were observed on a specimen of *Leonaspis rattei*—an odontopleurid from the Ludfordian Black Bog Shale, NSW (Bicknell & Smith, 2021, fig. 3a). These abnormal spine bases were attributed to fluctuating asymmetry—“random and uncorrelated deviations in the expression of normally bilateral characters” (Smith, 1998, pg. 99) indicating irregularities during the developmental processes. Although a more thorough examination of the Odontopleuridae is needed, these abnormal structures may be more common than previously considered.

Abnormal spines been observed in modern decapod crustaceans (Rasheed, Mustaquim & Khanam, 2014; İlkyaz & Tosunoğlu, 2019; Waiho, Ikhwanuddin & Fazhan, 2022) and horseshoe crabs (Bicknell & Pates, 2019; Bicknell et al., 2022b). The majority of these spines are associated with a larger injury and have therefore been attributed to complicated moulting or failed predation. However, in the rare situations where there is no evidence for injuries, possible genetic malfunctions have been presented to explain these spines (İlkyaz & Tosunoğlu, 2019). It seems possible that trilobites with a large number of spines may have experienced malfunctions in a similar fashion to modern, spine-bearing arthropods.

The distribution of *Odontopleura* (*Sinespinaspis*) *markhami* specimens in bivariate space illustrates that most abnormal specimens are located within the second largest size grouping. This could be interpreted as evidence for an increased frequency of abnormal spines within *O.* (*S.*) *markhami* during later growth stages. However, this pattern of increased specimens is influenced by the limited sampling from other size groups and the lack of a complete ontogenetic sequence of the species. As such, the presence of abnormal specimens in all developmental stages cannot be discounted. To shed more light on the presence of abnormal spines within *O.* (*S.*) *markhami,* more specimens, and ideally a complete development sequence, are needed. Further, examining abnormality patterns within other odontopleurid species, and trilobites more broadly, using a population-based approach will uncover generalized patterns across the clade’s extensive evolutionary history. However, such a collation of data was far beyond the scope of the present paper and represents important future directions for understanding abnormal specimens within trilobite populations.

Considering the record of abnormal Silurian trilobites from all parts of the globe (Table 1) most abnormal specimens record developmental complications and teratological recovery from substandard moulting (Bicknell & Smith, 2021), with rare examples of pathologies (De Baets et al., 2021). However, for the larger (>4 cm length) Silurian trilobites, such as *Arctinurus boltoni*, *Calymene niagarensis*, and *Dalmanites limulurus* from the Wenlock (Sheinwoodian) Rochester Formation, abnormalities include the removal of large exoskeletal sections (Babcock, 1993b; Whiteley, Kloc & Brett, 2002; Chinnici & Smith, 2015; Bicknell, Paterson & Hopkins, 2019). These record failed predation, as opposed to moulting complications (Chinnici & Smith, 2015; Bicknell, Paterson & Hopkins, 2019), especially as these taxa lack elongated pleural spines that would have complicated moulting (Conway Morris & Jenkins, 1985; Bicknell & Pates, 2020). The size of the species may therefore play a fundamental role in whether trilobite groups are targeted for predation. Indeed, Cambrian trilobites represented some of the largest prey items in the period and likely were targeted as food items (Bergström & Levi-Setti, 1978; Holmes, Paterson & García-Bellido, 2020; Bicknell et al., 2022a). The same is applicable for large, injured Ordovician species (Bicknell et al., 2022c; Bicknell et al., 2022d). As such, by the Silurian, other prey items (such as eurypterids) may have been preferred and only in select paleoecosystems were larger trilobite taxa subject to higher predation pressure. Alternatively, smaller trilobite species were completely consumed during predation, removing evidence from the fossil record. One possible means of testing this is to examine shelly coprolites from Silurian-aged deposits for trilobite fragments. Such an assessment may shed light on whether the bias for larger injured trilobites is a genuine biological signal, or the result of survivorship bias.

##  Supplemental Information

10.7717/peerj.14308/supp-1 Supplemental Information 1 Measurement data from *Odontopleura* (*Sinespinaspis*) *markhami* examined in Fig. 6 Includes data on whether the specimens were abnormal and the proposed size groupings presented in Fig. 6.

## Additional Information and Declarations

### Data Availability

The following information was supplied regarding data availability: The raw data is available in the Supplementary File.
